# Four novel mutations in the lactase gene (*LCT*) underlying congenital lactase deficiency (CLD)

**DOI:** 10.1186/1471-230X-9-8

**Published:** 2009-01-22

**Authors:** Suvi Torniainen, Roberta Freddara, Taina Routi, Carolien Gijsbers, Carlo Catassi, Pia Höglund, Erkki Savilahti, Irma Järvelä

**Affiliations:** 1 Department of Medical Genetics, University of Helsinki, Haartman Institute, Helsinki, Finland; 2 Neonatal Intensive Care Division, "G. Salesi" Children Hospital, Ancona, Italy; 3 Department of Paediatrics, Turku University Hospital, Turku, Finland; 4 Juliana Children's Hospital/Haga Teaching Hospital, The Hague, The Netherlands; 5 Department of Paediatrics, Università Politecnica delle Marche, Ancona, Italy; 6 Helsinki University Hospital, Laboratory Services, Helsinki, Finland; 7 Hospital for Children and Adolescents, University of Helsinki, Helsinki, Finland

## Abstract

**Background:**

Congenital lactase deficiency (CLD) is a severe gastrointestinal disorder of newborns. The diagnosis is challenging and based on clinical symptoms and low lactase activity in intestinal biopsy specimens. The disease is enriched in Finland but is also present in other parts of the world. Mutations encoding the lactase (*LCT*) gene have recently been shown to underlie CLD. The purpose of this study was to identify new mutations underlying CLD in patients with different ethnic origins, and to increase awareness of this disease so that the patients could be sought out and treated correctly.

**Methods:**

Disaccharidase activities in intestinal biopsy specimens were assayed and the coding region of *LCT *was sequenced from five patients from Europe with clinical features compatible with CLD. In the analysis and prediction of mutations the following programs: ClustalW, Blosum62, PolyPhen, SIFT and Panther PSEC were used.

**Results:**

Four novel mutations in the *LCT *gene were identified. A single nucleotide substitution leading to an amino acid change S688P in exon 7 and E1612X in exon 12 were present in a patient of Italian origin. Five base deletion V565fsX567 leading to a stop codon in exon 6 was found in one and a substitution R1587H in exon 12 from another Finnish patient. Both Finnish patients were heterozygous for the Finnish founder mutation Y1390X. The previously reported mutation G1363S was found in a homozygous state in two siblings of Turkish origin.

**Conclusion:**

This is the first report of CLD mutations in patients living outside Finland. It seems that disease is more common than previously thought. All mutations in the *LCT *gene lead to a similar phenotype despite the location and/or type of mutation.

## Background

Congenital lactase deficiency (CLD [MIM 223000]) is a severe form of lactase deficiency in which lactase activity is very low or absent in the intestinal wall from birth [1,2]. The main symptoms are watery diarrhoea in the first days of life after ingestion of lactose that leads to severe dehydration and loss of weight gain if untreated. Symptoms can be avoided and patients can have normal growth and development by changing to a lactose-free diet. The activities of other disaccharidases and the histological structure of the epithelium of the small intestine are normal [3,4]. Most CLD cases have been found in Finland where the disorder is enriched due to a founder effect and genetic drift [2,5]. This is in contrast to adult-type hypolactasia (where lactase activity declines after weaning) that is common all over the world [6-8]. Since the reports by Holzel et al. [1,9], there has been a lack of published data about CLD in countries other than Finland.

Mutations in the lactase gene (*LCT *[GeneID: 3938]) have recently been identified to underlie CLD in Finnish families [10]. Of the five mutations, Y1390X was the founder mutation present in 90% of the disease alleles. In addition, four other family specific mutations, S1666fsX1722, S218fsX224, G1363S and Q268H were identified in the Finnish population. However, no mutations have been found outside Finland. In order to further characterize the spectrum of mutations we have sequenced the coding region and splice sites of the *LCT *gene in five patients originating from Italy, Finland and Turkey, with clinical features compatible with CLD.

## Methods

### Case 1

A male infant of unrelated, healthy parents of Italian origin with unremarkable family histories was born at term after an uneventful pregnancy with a birth weight of 3180 g. At the age of 10 days, he was admitted to the neonatology intensive care unit because of metabolic acidosis. On admission, his weight was 2774 g and he showed watery diarrhoea on breast feeding. Clinitest^® ^on stools was strongly positive, reducing sugars higher than 20 g/dl and stool pH was low (pH5). Diarrhoea promptly stopped after starting parenteral rehydration and stopping oral feeding. After seven days a further attempt to breast-feed this baby, led again to osmotic diarrhoea and weight loss. Stools quickly became normal after putting the child on a lactose-free, cow's milk protein-based formula, where the child remained well. At age 3 months his weight was 5890 g (50^th ^centile). An oral lactose tolerance test with 2 g/kg of lactose showed no increase in glucose blood levels within 120 minutes and was followed by watery diarrhoea within a few hours. His faeces were positive for reducing sugars, which was shown to be lactose by high-performance liquid chromatography (HPLC). The child was discharged on a lactose-free, soy protein-based formula and remained well on a lactose-free diet. At age 12 months, his weight was 9860 g (50^th ^centile). A small intestinal biopsy specimen was taken using the Watson capsule. His small intestinal morphology was normal on standard histological examination, as far as the villous height, crypt depth and intraepithelial lymphocyte count were concerned. Lactase activity measured by the Dahlqvist method [11] at the Scientific Laboratory of the Hospital for Children and Adolescents, University of Helsinki was very low 4 U/g/protein, while values of other disaccharidases were normal (data not shown). At the last follow-up visit (age 15 months) this baby was perfectly healthy on a lactose-free diet.

### Case 2

The Finnish patient was the first child of the healthy parents. Because of pre-eclampsia and intrauterine growth failure, a Caesarean section was performed after 35 weeks of gestation. After birth, oral and intravenous nutrition were started. On breast milk, the infant needed high amounts of iv glucose, and had watery stools. When put on hydrolyzed, lactose free formula, the diarrhoea stopped and iv nutrition could be stopped at the age of 2 weeks. At 4 weeks of age, he was transferred to breast milk and sent home. After one week on breast milk at home, he was taken back to the hospital. Loss of weight and severe acidosis was found in the patient. After iv fluid therapy, he was put back on the hydrolyzed formula and based on the disappearance of symptoms, he was suspected to have congenital lactase deficiency (CLD). At the age of 6 months, a jejunal specimen for morphological studies, and disaccharidase activity measurements was obtained by a suction biopsy device [11]. The morphology of his duodenum was quite normal; the activity for maltase and sucrase were normal (data not shown). Lactase activity was 8 U and the lactase/sucrase ratio was 0.13. The infant was considered to have CLD and has been well on a lactose-free diet.

### Case 3

A five-week-old Finnish boy was referred to the paediatric outpatient clinic of Turku University Hospital because of poor weight gain and failure-to-thrive. He was born at term after an uneventful pregnancy as the second child of healthy parents with a birth weight of 3810 g. He received breast milk until two weeks of age, then he was put on a regular, adapted, infant formula (Tutteli^®^, Valio Ltd, Finland) but had poor weight gain and watery diarrhoea from the first days of life. At the age of 5 weeks, his weight was 150 grams below the birth weight though he ate reasonably big amounts of infant formula (900 ml/d). He showed metabolic acidosis (base excess was -12.3, pH 7.28, HCO3 14.9) and plasma sodium was high 165 mmol/l. In hospital, the values were corrected within a day with iv infusions. His faecal elastase was found to be normal, but his stools contained high amounts of reducing sugars. Because CLD was suspected, nutrition with lactose-free whey-based, hydrolyzed formula (Pepti-Junior^®^, Nutricia, Netherlands) was started. The diarrhoea ceased during the first day and his weight gain improved remarkably. In specimen taken by gastroduodenoscopy, the morphology of his upper gastrointestinal tract was normal, but a very low activity of lactase was found in the duodenal specimen (because of the small size of the specimen, the laboratory did not give any exact lactase value, but they could not detect any activity) [11]. Later, at the age of 11 months lactose malabsorption was verified by a positive breath hydrogen test. Growth was normal on a lactose free, whey-hydrolysate formula (Pepti-Junior). After the age 12 of months he was put on a lactose-free regular milk and he showed no gastrointestinal symptoms.

### Case 4 and 5

Case 4, a four-day-old Dutch boy was admitted to the hospital because of crying, irritability, a low grade fever and refusing to eat starting a few hours earlier. He was born at term to consanguineous parents (first cousins) of Turkish origin after an uneventful pregnancy. He was breast-fed and had yellow watery stools without blood or mucus from birth. At presentation he was malnourished, distressed and irritated. His weight was 8% below birth weight. He was suspected of having septicemia, but cultures appeared to be negative. His plasma sodium was 152 mmol/l, base excess -12.8 mmol/l and faecal pH 5.4. Discontinuation of breastfeeding resulted in normalization of stools within 18 hours. A carbohydrate free formula was tolerated well; with addition of glucose to this feeding he had normal stools, after adding lactose to the feeding he again had several fluid stools and vomited. He was discharged with a soy formula, on which he grew perfectly. When he was 3 months and 10 months old duodenoscopies were done; biopsies showed normal villi. At the age of three months lactase activity was 1 U/g/protein (reference values 2.8–6.7 U/wet weight). Activities of other disaccharidases measured at the Erasmus Medical Centre Rotterdam [11] and alkaline phosphatase were normal (data not shown). At the age of 10 months his lactase was < 1 U/g/protein (ref 38.5 ± 12.5), again with normal maltase and sucrase activities (data not shown) (disaccharidase activities were measured at the Academic Medical Centre Amsterdam).

Case 5, the younger sister of case 4 was born at term after an uneventful pregnancy. On day 3 after breastfeeding she started to suffer from diarrhoea; on admission after some hours her weight was 13% below birth weight. Soy formula was started and her stools became more firm after one week. She did very well on the soy formula. At age 11 months a duodenoscopy was performed, which showed completely normal villi. Her lactase was 0 U/g/protein (reference values 2.8–6.7 U/wet weight), with normal activities of other disaccharidases (data not shown) (disaccharidase activities measured at the Academic Medical Centre Amsterdam).

### Control subjects

The control subjects were matched with the nationality of the patients. Therefore, the control subjects consisted of 98 Finnish and 101 Italian control samples.

#### Ethics

The study protocol has been approved by the Ethics Committee of Helsinki University Hospital and performed in accordance with the Declaration of Helsinki. The parents' written informed consent was obtained.

### PCR-sequencing

DNA was isolated from peripheral blood of the patients and their parents and amplified by polymerase chain reaction (PCR) covering the 17 exons and splice sites of the *LCT *gene as described previously [10]. Primer sequences are available on request from the authors. The size of the PCR product was verified by 1.5% agarose gel electrophoresis with ethidium bromide. PCR products were purified using 2.5 U of Shrimp Alkaline Phosphatase (USB) and 5 U of Exonuclease I (New England Biolabs) at 37°C for 45 min and at 80°C for 15 min. For sequencing the BigDye 3.1 terminator (Applied Biosystems) was used according to the manufacturer's instructions. The sequencing reaction was as follows: at 96°C for 1 min, then 25 cycles at 96°C for 10 s, at 55°C for 5 s and at 60°C for 4 min. The sequencing reaction followed purification by Millipore Multiscreen plates (Millipore, USA) with Sephadex G-50 Superfine sepharose (Amersham Biosciences, Sweden), electrophoresis by ABI 3730 DNA Analyzer (Applied Biosystems) and base calling by Sequencing Analysis 5.2 software (Applied Biosystems). The obtained sequence was analyzed using Sequencher 4.1.4 software (Gene Codes, USA).

### Mutation prediction

The ClustalW alignment program was used to study conservation of the mutation sites among different species [12]. To analyze the probability of specific amino acid substitutions Blosum62 (BLOck SUbstitution Matrix) scores were obtained [13]. The possible functional impact of an amino acid change was predicted by the PolyPhen (Polymorphism Phenotyping) program [14], which makes predictions based on sequence annotation and alignment as well as structural information, SIFT (Sorting Intolerant From Tolerant) [15] and PANTHER PSEC [16] that use sequence homology for prediction. SIFT gives a prediction for whether the substitution is tolerated or not and PATNTHER gives a substitution PSEC (position specific evolutionary conservation) score.

## Results

### Case 1

**Figure 1:**
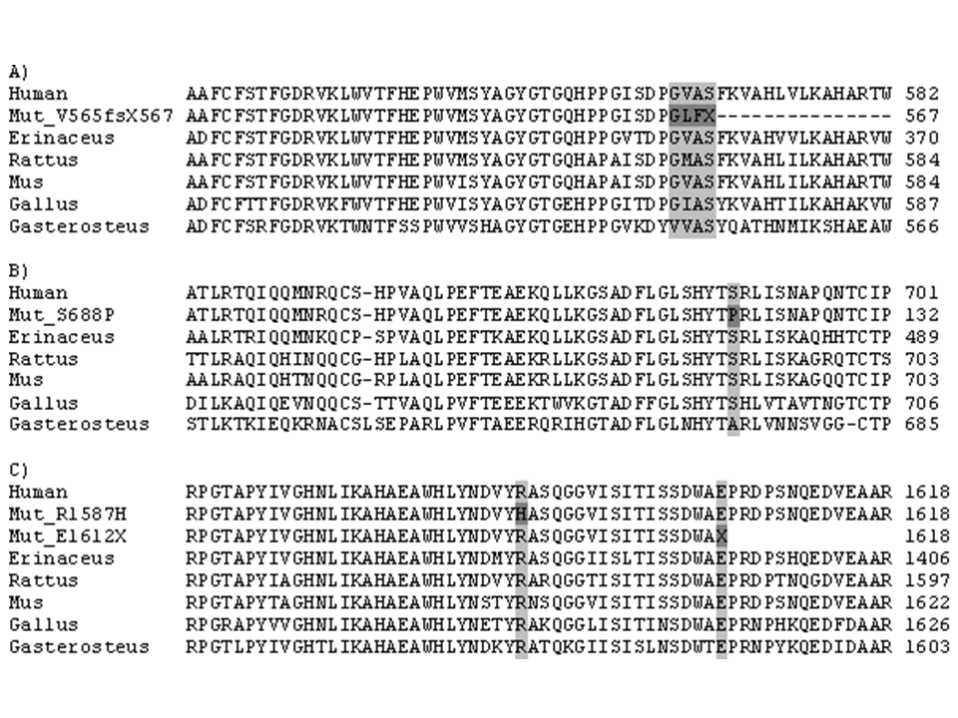
**Conservation of mutations sites**. ClustalW alignment of human LPH protein and LPH proteins from other species showing the conservation of the region for mutations V565fsX567 (A), S688P (B), R1587H (C) and E1612X (C).

Two nucleotide substitutions in a heterozygous form in the *LCT *gene were identified in the Italian patient: c.2062T > C in exon 7 leading to a substitution of serine to proline (S688P) and c.4834G > T in exon 12 leading to glutamic acid to change to a premature stop codon (E1612X) (Additional file 1). The mother of the patient was shown to be a carrier of the mutation S688P and the father a carrier of the mutation E1612X. No substitutions were found in 101 Italian control subjects for E1612X and 97 for S688P suggesting that they are rare events. Using ClustalW analysis, both mutations were located in the conserved site (Figure 1). The Blosum62 score for substitution from serine to proline is -1 (range from -4 to +11; low to high probability of substitution) [17]. The effect of mutation S688P was predicted to be possible damaging by PolyPhen (position-specific independent counts; PSIC score difference 1.773) (score > 1.7 considered damaging) [18] and not tolerated by SIFT. From PANTHER PSEC results could not be obtained.

### Case 2

Deletion of five bases c.1692-1696delAGTGG in exon 6 leading to frameshift mutation V565fsX567 (Additional file 1) was found in Finnish patient heterozygous for the founder mutation Y1390X [10]. The deletion was located in the conserved region of the *LCT *gene (Figure 1) and was predicted to lead to truncated protein due to a change in the reading frame. Unfortunately parental DNA was not available for the analysis. The deletion was not found in 98 anonymous Finnish blood donors.

### Case 3

The Finnish patient was first shown to be heterozygous for the founder mutation Y1390X [10]. Sequencing of the *LCT *gene resulted in the identification of substitution c.4760G→A in exon 12 which changes arginine to histidine (R1587H) (Additional file 1). The mother was a carrier of the founder mutation and the father a carrier of the novel substitution. The R1587H was not identified in 98 anonymous Finnish blood donors. Using the ClustalW alignment program the amino acid was shown to be conserved (Figure 1). The Blosum62 score for substitution from arginine to histidine is 0. The effect of mutation R1587H was predicted to be possible damaging by PolyPhen (PSIC score difference 1.820) and not tolerated by SIFT. The PANTHER subPSEC score was -4.41655 (cut off point for deleterious mutation is < -3) [19].

### Case 4 and 5

The previously known substitution c.4087G > A in exon 9 leading to an amino acid change from glycine to serine (G1363S) (Additional file 1) was identified in a homozygous form in both siblings. Both parents were shown to be carriers of the same mutation.

## Discussion

Here we report four new mutations in the *LCT *gene underlying congenital lactase deficiency (CLD). The mutations were identified in compound heterozygous form in three patients (Italian and Finnish) with characteristic features of CLD. There are three kinds of evidence that these mutations lead to CLD 1) the substitutions change the amino acid sequence of a protein in a conserved region of the LPH protein, 2) substitutions were predicted to be damaging by several prediction methods and 3) healthy control subjects lack these mutations. We also found mutation G1363S in a homozygous form in two siblings of Turkish origin. This mutation has earlier been reported in one Finnish CLD patient in eastern (near the Russian border) Finland suggesting that the mutation may have been brought to Finland from the east [10]. This is the first time that CLD cases and the underlying mutations have been identified outside Finland.

**Figure 2:**
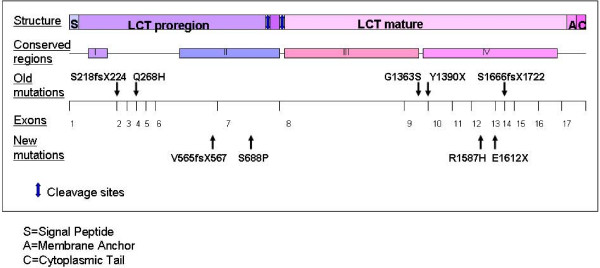
**Schematic presentation of the *LCT *gene and locations of the mutations**. Cleavage sites are R734/L735 and R867/A868.

Mutations E1612X and R1587H are located in the conserved region IV of the mature *LCT *that encodes lactase activity (Figure 2) [20-22]. Mutation E1612X leads to a truncated protein. In substitution R1587H both amino acids are polar, basic amino acids. However, arginine always has a positive charge and a role in the maintenance of protein overall charge balance. Therefore the substitution to histidine (which charge depends on environment) may disturb the overall charge. Substitution S688P and deletion V565fsX567 are located in the region II of the pro-LPH that has been shown to have a role as an intramolecular chaperone in the folding of the LPH-protein (Figure 2) [23,24]. Deletion V565fsX567 leads to truncated protein after a few amino acids. A change from a non-aromatic polar serine to cyclic and non-polar proline causes a remarkable change in the chemical and physical properties of the protein.

All four novel mutations were examined by several prediction programs [17]. Although the Blosum62 matrix does not take into account the conservation of the protein region, the results showed that there is low probability of both substitutions (serine to proline and arginine to histidine) of occurring in the functional protein. In PolyPhen the prediction was based on PSIC score (logarithmic ratios of likelihood of a studied amino acid occurring at a particular site to the likelihood of same amino acid occurring at any site) [18]. Since multiple alignment profile scores provide a major contribution to the prediction, it remains reasonably reliable even without the missing 3-D information of LPH. [18]. Using PolyPhen both substitutions (R1587H and S688P) were considered to be pathogenic. The results obtained with SIFT or PANTHER PSEC were in line with the results above.

Taken together, a total of nine mutations are known to underlie CLD to date. The mutations are quite evenly distributed covering both the pro region and the mature LPH (Figure 2). Location of mutations does not affect the severity of the phenotype. In contrast, all mutations lead to a severe phenotype. The phenotype of CLD is in striking contrast with the phenotype of adult-type hypolactasia, associated with the normal down-regulation of lactase activity with mild or absent symptoms. It is noteworthy that in these two conditions, the values of lactase activity are partially overlapping in spite of their having different underlying molecular mechanism [2,5-8,10]. However symptoms are much more severe in CLD.

After the identification of the molecular background of CLD, genetic testing has been offered in Finland. Interestingly, nine novel CLD cases were identified in 2006 and two in 2007. All patients were diagnosed at the first weeks of age based on severe watery diarrhoea and loss of weight. Nine of them were homozygous for the major mutation Y1390X further confirming the original results that only one major mutation is enriched in the Finnish population [10]. Two patients were heterozygous for the founder mutation. In both of them (case 2 and 3) new mutations were reported here. Earlier, CLD has been estimated to be very rare with a frequency of 1:60 000 newborns in Finland [5]. The figures presented here suggest that CLD may be more common than previously estimated. CLD should be suspected in neonates with severe diarrhoea which starts after introduction of milk feeding. A high concentration of lactose is present in liquid faeces and may easily be identified. The diarrhoea is cured within hours by substituting milk with a lactose-free formula. Later, CLD may be diagnosed by either genetic testing or by studying disaccharidase concentrations in the duodenal mucosa.

## Conclusion

Four new mutations that underlie CLD were identified, of these, two are the first mutations found in continental Europe. The location of mutation in *LCT *does not seem to affect the severity of the disease. CLD seems to be more common than previously assumed.

## Abbreviations

BLOSUM: block substitution matrix; CLD: congenital lactase deficiency; HPLC: high-performance liquid chromatography; LPH: lactase phlorizin-hydrolase; *LCT*: lactase coding gene; PolyPhen: polymorphism phenotyping; PSEC: position specific evolutionary conservation; PSIC: position-specific independent counts; SIFT: sorting intolerant from tolerant

## Competing interests

The authors declare that they have no competing interests.

## Authors' contributions

ST carried out sequencing, analysis of results, predictions of mutations and drafted the manuscript. RF diagnosed the Italian patient and drafted the manuscript. TR diagnosed the Finnish patient, provided description of Finnish patient and participated in overall drafting. CG diagnosed the Turkish origin patients, provided descriptions of these patients and participated in overall drafting. CC diagnosed the Italian patient and drafted the manuscript. PH participated in designing the study, provided incidence rates of CLD and the original information about patients with unknown mutations in Finland. ES diagnosed the other Finnish patient, carried out disaccharidase assays of Finnish and Italian patients and provided his expertise in drafting the manuscript. IJ conceived the idea of the study, coordinated the study and helped to draft the manuscript. All authors read and approved the final manuscript.

## Pre-publication history

The pre-publication history for this paper can be accessed here:

http://www.biomedcentral.com/1471-230X/9/8/prepub

## Supplementary Material

Additional file 1 **The mutations in the LCT gene found in the patients of this study.** Data represents the lactase activities and mutations in DNA and protein level in patients of this study. Primer sequences used in identification of mutations are also provided. Click here for file
