# Supplementary material for: Four novel mutations in the lactase gene (LCT) underlying congenital lactase deficiency (CLD)
Source: BMC Gastroenterol. 2009 Jan 22;9:8. doi: 10.1186/1471-230X-9-8 (PMC2635369; doi:10.1186/1471-230X-9-8)
Supplement: Additional file 1 — The mutations in the LCT gene found in the patients of this study. Data represents the lactase activities and mutations in DNA and protein level in patients of this study. Primer sequences used in identification of mutations are also provided. [file 1471-230X-9-8-S1.doc]

**Additional file 1. The mutations in the *LCT* g**ene found in the patients of this study.

| Case number | DNA mutation | Protein mutation | Exon | Primer sequences | Genotype | Ethnic origin | Lactase activity (U/g/  protein |
| --- | --- | --- | --- | --- | --- | --- | --- |
| 1 | c.4834G>T^a^ | E1612X ^a^ | 12 | F: 5’-GCAGGGACTAACAATCTCAGT-3’  R: 5’-AGGCTGGAAGGAAAGATGGAC-3’ | Heterozygote | Italian | 4 |
|  | c.2062T>C^a^ | S688P ^a^ | 7 | F: 5’-GTGCTGATGTTGTCAATATTGAC-3’  R: 5’-ACTTTGAAGACTTGGAGAAGTAC-3’ | Heterozygote |  |  |
| 2 | c.4170T>A | Y1390X | 9 | F: 5’-ATGGAGGTCCTGACTCCTGGT-3’  R: 5’-GTGTGAAGAAGCCAGGCTGTC-3’ | Heterozygote | Finnish | 8 |
|  | c.1692_1696delAGTGG^a^ | V565fsX567^a^ | 6 | F: 5’-GCGTTGCCTACTACAACAAGC-3’  R: 5’-CTGGTCTCGAACTCCTGGAC-3’ | Heterozygote |  |  |
| 3 | c.4170T>A | Y1390X | 9 | F: 5’-ATGGAGGTCCTGACTCCTGGT-3’  R: 5’-GTGTGAAGAAGCCAGGCTGTC-3’ | Heterozygote | Finnish | 0 |
|  | c.4759G>A^a^ | R1587H ^a^ | 12 | F: 5’-GCAGGGACTAACAATCTCAGT-3’  R: 5’-AGGCTGGAAGGAAAGATGGAC-3’ | Heterozygote |  |  |
| 4 | c.4087G>A | G1363S | 9 | F: 5’-ATGGAGGTCCTGACTCCTGGT-3’  R: 5’-GTGTGAAGAAGCCAGGCTGTC-3’ | Homozygote | Turkish | 1 |
| 5 | c.4087G>A | G1363S | 9 | F: 5’-ATGGAGGTCCTGACTCCTGGT-3’  R: 5’-GTGTGAAGAAGCCAGGCTGTC-3’ | Homozygote | Turkish | 0 |

^a^ New mutation
